# An optimized method to obtain high-quality RNA from different tissues in *Lilium davidii* var. *unicolor*

**DOI:** 10.1038/s41598-022-06810-7

**Published:** 2022-02-18

**Authors:** Chunlei Wang, Xuemei Hou, Nana Qi, Changxia Li, Yanyan Luo, Dongliang Hu, Yihua Li, Weibiao Liao

**Affiliations:** College of Horticulture, Gansu Agricultural University, Lanzhou, 730070 People’s Republic of China

**Keywords:** Physiology, Plant sciences

## Abstract

The high quality, yield and purity total RNA samples are essential for molecular experiments. However, harvesting high quality RNA in *Lilium davidii* var. *unicolor* is a great challenge due to its polysaccharides, polyphenols and other secondary metabolites. In this study, different RNA extraction methods, namely TRIzol method, the modified TRIzol method, Kit method and cetyltrimethylammonium bromide (CTAB) method were employed to obtain total RNA from different tissues in *L. davidii* var. *unicolor*. A Nano drop spectrophotometer and 1% agarose gel electrophoresis were used to detect the RNA quality and integrity. Compared with TRIzol, Kit and CTAB methods, the modified TRIzol method obtained higher RNA concentrations from different tissues and the A260/A280 ratios of RNA samples were ranged from 1.97 to 2.27. Thus, the modified TRIzol method was shown to be the most effective RNA extraction protocol in acquiring RNA with high concentrations. Furthermore, the RNA samples isolated by the modified TRIzol and Kit methods were intact, whereas different degrees of degradation happened within RNA samples isolated by the TRIzol and CTAB methods. In addition, the modified TRIzol method could also isolate high-quality RNA from other edible lily bulbs. Taken together, the modified TRIzol method is an efficient method for total RNA isolation from *L. davidii* var. *unicolor*.

## Introduction

Lanzhou lily (*Lilium davidii* var*. unicolor*), an important economic crop, is the only edible sweet lily in China. It is an endemic species that is only suitable at the altitude of 2000–2600 m, resulting in a narrow distribution in Gansu Province, western China^1^. Bulbs of the *L. davidii* var*. unicolor* are one of the main traditional Chinese medicines for several centuries, and its flowers also have ornamental value^2,3^. In recent years, more and more studies focused on bulbs of *L. davidii* var*. unicolor*, such as bulblets formation and development, and dormancy of bulbs. High levels of starches, proteins, fats, celluloses, saponins, colchicines, and polysaccharides in scales of the bulb can be used as potential biomarkers for bulb quality^4,5^. The starch and sucrose metabolism is the major pathway in the scales of this *Lilium* species^6,7^. In addition, the content of polysaccharides in *Lilium* roots is also very high. During the study concerning growth and development of *Lilium*, works on the physiological and molecular aspects is necessary, whereas it is difficult to obtain high quality RNA to support molecular biology techniques. Polysaccharides and polyphenols limit nucleotide purification and precipitation due to their co-precipitation with the endogenous RNA^8,9^. Thus, obtaining high-quality total RNA for many molecular analyses from scales and other tissues of *Liliu*m is difficult due to the high level of various metabolites. Therefore, the optimized method to isolate total RNA effectively from different tissues of *Lilium* should be explored.

Over the past several decades, commercial plant kits play an important role in plant molecular studies, but extracting RNA with high quality and quantity from plants rich in polysaccharides remains a challenge^10^. Moreover, Kit is used to extract high quality and integrated RNA, which is not only depending on plant species, genotype and tissue type, but also related to the content of polyphenols, polysaccharides and proteins^10,11^. As for researches of lily bulbs containing multiple polyphenols, polysaccharides, proteins and lipids, removing these metabolites is a vital procedure during RNA isolation^12,13^. By now, several RNA extraction methods have been reported to be available for *Brassica* plants^12^, *Pistacia vera* L.^14^, oil seeds^15^ and cassava tubers^16^. Also, some methods emerged to isolate total RNA from *L. davidii* var. *unicolor*, in which cetyltrimethylammonium bromide (CTAB) method was reported to be more suitable for RNA extraction in the bulb^17,18^. In addition, TRIzol method and RNA extraction Kit were employed to separate RNA from leaf and bulb of Lanzhou lily^2,19–21^. Nevertheless, few protocols are suitable for isolating total RNA from different tissues of *Lilium*, especially from different parts of the scales. Despite the rapid advances in genetic engineering and transgenic technology of model plants, the genomic database of *Lilium* is needed to be further improved. Therefore, obtaining integrity, high quality and yield total RNA from *Lilium* is an essential condition for functional genomics research.

In this study, different RNA extraction methods, including the modified TRIzol method, TaKaRa MiniBEST Plant RNA Extraction Kit (Kit) method and CTAB method, were used to extract total RNA from different tissues of *L. davidii* var. *unicolor* (root, stem, leaf, top-inner scales, middle-inner scales, basal-inner scales, top-middle scales, middle-middle scales, basal-middle scales, top-external scales, middle-external scales and basal-external scales). In addition, the quality and yield of RNA obtained by TRIzol method and the modified TRIzol method were compared with each other within different tissues of *L. davidii* var. *unicolor*, and within different edible bulbs. A Nano drop spectrophotometer and 1% agarose gel electrophoresis were used to assess the quality of the extracted RNA samples. Here, we concluded that the modified TRIzol method could be applicable to gain high quality and quantity total RNA in different tissues of *L. davidii* var*. unicolor* containing polyphenols, polysaccharides, proteins and lipids.

## Results

### RNA samples with higher quality and yield were extracted by the modified TRIzol method from root, stem and leaf in *L. davidii* var. *unicolor*

Three methods were used for extracting RNA samples from root, stem and leaf tissues of *L. davidii* var*. unicolor*, including the modified TRIzol method, Kit method and CTAB method. In *Lilium* root, RNA samples obtained from the modified TRIzol method showed that the A260/A230 and A260/A280 ratios were closed to 1.5 and 2.1, and RNA samples gained from the CTAB method showed higher ratios (ratios of A260/A230 and A260/A280: 1.9 and 2.2, respectively) (Table 1). However, RNA prepared from root with the Kit method showed lower absorbance ratios (ratios of A260/A230 and A260/A280: 1.0 and 2.0, respectively). Among these methods, the highest RNA concentration was obtained by using the modified TRIzol method (565.39 ng μL^−1^) (Table 1). For stem tissue of *Lilium* as shown in Table 1, the A260/A280 and A260/A230 ratios of the RNA samples extracted by the Kit and CTAB methods were less than 1.0 and 1.8, respectively, indicating that the extracted RNA was of low purity. However, the modified TRIzol method resulted in higher A260/A230 ratio (1.0), A260/A280 ratio (1.97) and yield (165.69 ng μL^−1^). Therefore, high-quality total RNA could be isolated from *Lilium* stem by using the modified TRIzol method. When RNA was extracted from *Lilium* leaf using the Kit and CTAB methods, the spectrophotometric results showed that the A260/A230 ratios of RNA ranged from 0.91 to 1.07, and the A260/A280 ratios ranged from 1.69 to 2.01, indicating that the RNA samples were contaminated by proteins, polyphenols and polysaccharides. However, when using the modified TRIzol method, the A260/A230 and A260/A280 ratios and the RNA concentrations were increased significantly (Table 1).

**Table 1:** Average purity and yield of total RNA extracted from root, stem and leaf of *Lilium davidii* var. *unicolor* using different methods.

Parts	260/230				260/280				Concentration (ng μL^−1^)			
	TRIzol	Modified TRIzol	Kit	CTAB	TRIzol	Modified TRIzol	Kit	CTAB	TRIzol	Modified TRIzol	Kit	CTAB
Root	1.37 ± 0.06b	1.51 ± 0.28ab	1.05 ± 0.15b	1.92 ± 0.03a	2.16 ± 0.00b	2.16 ± 0.01b	2.03 ± 0.04c	2.23 ± 0.00a	394.98 ± 6.65b	565.39 ± 9.67a	56.73 ± 8.77d	259.49 ± 13.80c
Stem	1.24 ± 0.07a	1.00 ± 0.24ab	0.43 ± 0.25b	0.98 ± 0.25ab	1.96 ± 0.01a	1.97 ± 0.02a	1.75 ± 0.10b	1.68 ± 0.07b	133.99 ± 0.44a	165.69 ± 27.65a	25.35 ± 7.92c	79.69 ± 3.80b
Leaf	1.37 ± 0.04a	1.28 ± 0.23a	1.07 ± 0.03a	0.91 ± 0.01a	2.05 ± 0.06a	2.02 ± 0.02a	2.01 ± 0.10a	1.69 ± 0.07b	422.43 ± 8.25a	227.85 ± 14.90b	69.45 ± 4.81c	50.27 ± 13.03c

In order to compare TRIzol method and the modified TRIzol method, the RNA samples obtained from root, stem and leaf by use of the TRIzol method were also analyzed. The A260/A230 and A260/A280 ratios of the RNA gained by the modified TRIzol method showed no difference with those of the TRIzol method. Nevertheless, the RNA concentrations of root and stem obtained from the TRIzol method (394.98 ng μL^−1^ and 133.99 ng μL^−1^, respectively) were significantly lower than that from the modified TRIzol method (Table 1). Taken together, the modified TRIzol method is the most efficient and reproducible method to extract high-quality total RNA from root, stem and leaf tissues of *L. davidii* var*. unicolor*.

**Figure 1:**
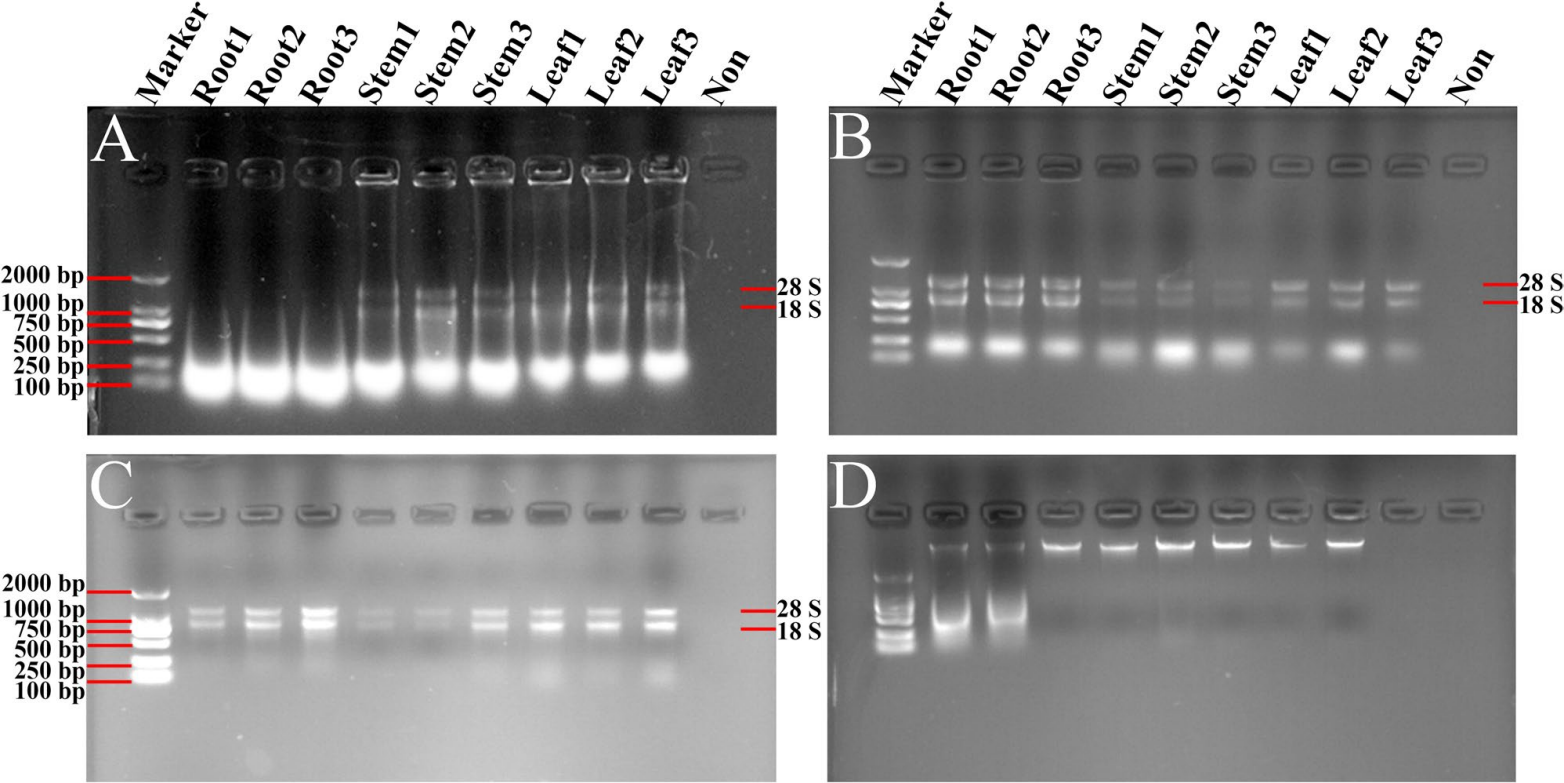
Gel images of total RNAs from root, stem and leaf tissues of *Lilium davidii* var. *unicolor* isolated using different protocols. The RNA samples extracted by TRIzol method (**A**), the modified TRIzol method (**B**), Kit method (**C**) and CTAB method (**D**) were separated and analyzed in 1% agarose gels, respectively. Then the gels were visualized and exposed by Gel Imager System in different fields, and pictures were taken, respectively. The order of samples in each gel lane is as follows: Lane 1: DL2000 DNA marker; Lanes 2–4 are RNA isolated from root of *Lilium*; Lanes 5–7 are RNA isolated from stem of *Lilium*; Lanes 8–10 are RNA isolated from leaf of *Lilium*; Lane 11: Loading buffer solution without RNA to serve as a control. 28S and 18S represent the location of 28S and 18S rRNA bands.

The integrity of isolated RNA samples was then tested by using 1% agarose gel electrophoresis. The RNA samples from *Lilium* root isolated by the TRIzol method showed obviously degradation, and the detected bands of 28S and 18S ribosomal RNA (rRNA) from stem and leaf were quite shallow (Fig. 1A). Moreover, the RNA samples gained from the modified TRIzol and Kit methods showed legible and integrated bands of 28S rRNA and 18S rRNA without obvious DNA contamination (Fig. 1B,C). However, the RNA samples obtained by the CTAB method showed visible chromosomal DNA bands, and the corresponding RNA bands were degraded seriously (Fig. 1D). To this end, the obtained total RNA by the modified TRIzol method showed high yield and quality as revealed by a high RNA integrity.

### The Kit method is the effective method in isolating RNA from inner scales of *L. davidii* var. *unicolor*

In inner scales of *L. davidii* var*. unicolor*, the absorbance spectra revealed that the A260/A230 ratios (ranged from 0.27 to 0.4) of RNA samples isolated by the modified TRIzol method were lower than that by the Kit (ranged from 1.96 to 2.06) and CTAB (ranged from 1.74 to 1.92) methods, but were higher than that by the TRIzol method (ranged from 0.19 to 0.22) (Table 2). Additionally, only the A260/A280 ratios of RNA segregated by the TRIzol method were below 2.00 (ranged from 1.85 to 1.95), and the A260/A280 ratios of RNA gained by the other three methods were ranged from 2.10 to 2.28 (Table 2). These results suggested that the RNA samples isolated by the Kit and CTAB methods contained lower protein, organic solvents, and other contaminants compared to the TRIzol and modified TRIzol methods. Moreover, the Kit method could obtain a higher RNA yield (ranged from 586.04 to 1025.19 ng μL^−1^) compared to the other methods, and quality of RNA gained by the TRIzol method (ranged from 131.31 to 401.52 ng μL^−1^) were the worst (Table 2).

**Table 2:** Average purity and yield of total RNA extracted from inner scales of *Lilium davidii* var. *unicolor* using different methods.

Parts	260/230				260/280				Concentration (ng μL^−1^)			
	TRIzol	Modified TRIzol	Kit	CTAB	TRIzol	Modified TRIzol	Kit	CTAB	TRIzol	Modified TRIzol	Kit	CTAB
T-inner	0.22 ± 0.00b	0.27 ± 0.03b	2.06 ± 0.00a	1.74 ± 0.22a	1.95 ± 0.00c	2.10 ± 0.01b	2.28 ± 0.01a	2.20 ± 0.07ab	276.2 ± 4.17c	293.23 ± 47.43c	586.04 ± 45.06a	418.80 ± 16.49b
M-inner	0.19 ± 0.00b	0.3 ± 0.03b	2.01 ± 0.04a	1.92 ± 0.06a	1.87 ± 0.00b	2.27 ± 0.05a	2.25 ± 0.02a	2.26 ± 0.02a	401.52 ± 8.8b	506.23 ± 122.91b	781.20 ± 29.16a	382.29 ± 31.59b
B- inner	0.2 ± 0.00b	0.4 ± 0.04b	1.96 ± 0.02a	1.81 ± 0.17a	1.85 ± 0.02b	2.21 ± 0.02a	2.26 ± 0.01a	2.19 ± 0.04a	131.31 ± 5.17c	609.70 ± 132.36b	1025.19 ± 44.44a	450.56 ± 62.34b

*T* The top part of the detected scales, *M* The middle part of the detected scales, *B* The basal part of the detected scales.

**Figure 2:**
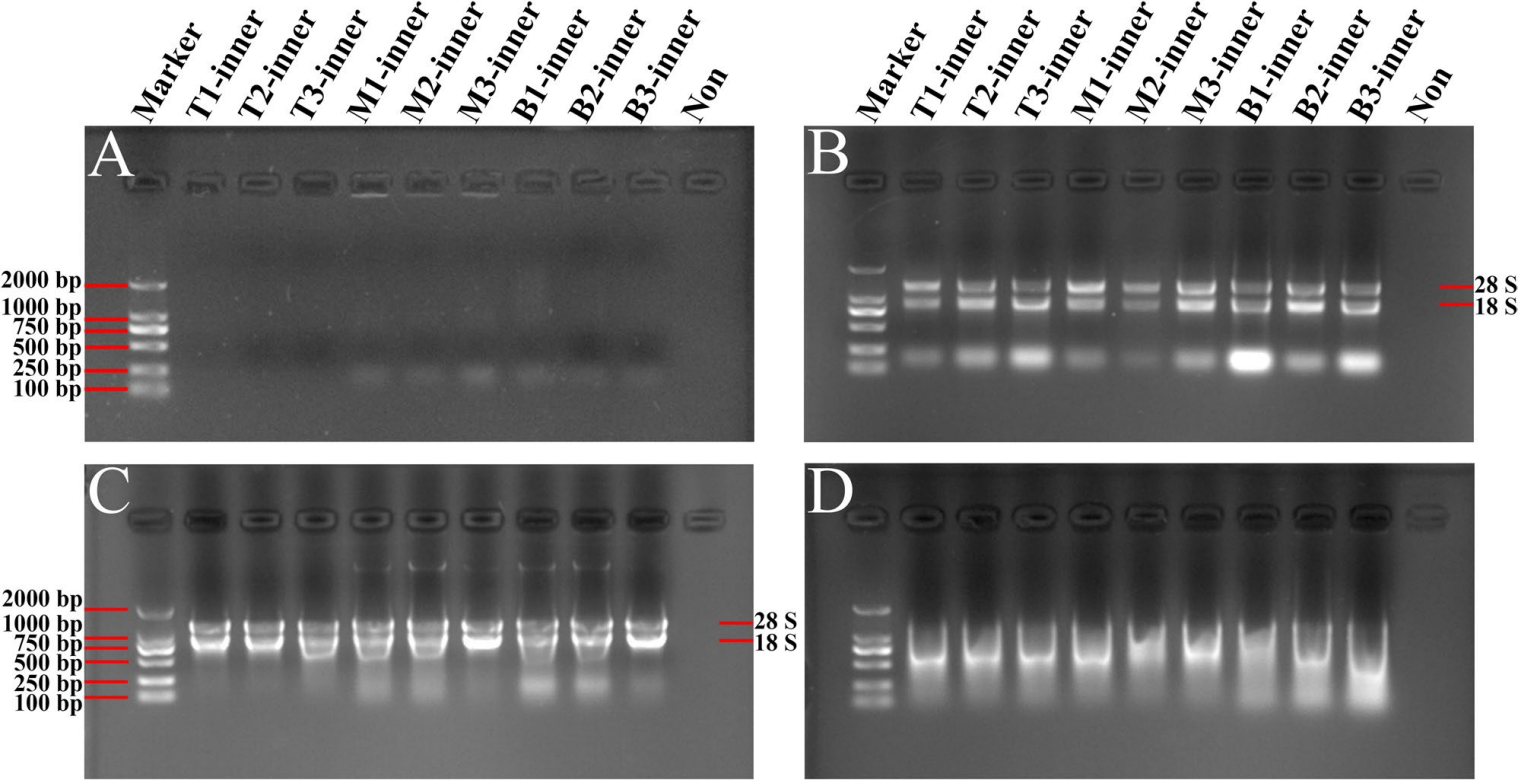
Gel images of total RNA from inner scales of *Lilium davidii* var. *unicolor* isolated using different protocols. The RNA samples extracted by TRIzol method (**A**), the modified TRIzol method (**B**), Kit method (**C**) and CTAB method (**D**) were separated and analyzed in 1% agarose gels, respectively. Then the gels were visualized and exposed by Gel Imager System in different fields, and pictures were taken, respectively. The order of samples in each gel lane is as follows: Lane 1: DL2000 DNA marker; Lanes 2–4 are RNA isolated from top scales of *Lilium*; Lanes 5–7 are RNA isolated from middle scales of *Lilium*; Lanes 8–10 are RNA isolated from basal scales of *Lilium*; Lane 11: Loading buffer solution without RNA to serve as a control. 28S and 18S represent the location of 28S and 18S rRNA bands.

As for 1% agarose gel electrophoresis analysis, we failed to detect any RNA bands with the RNA samples obtained by the TRIzol method (Fig. 2A). Interestingly, the RNA bands from the Kit and modified TRIzol methods were clear and intact, testifying that these RNA samples were not degraded (Fig. 2B,C). However, the separated RNA from the CTAB method was degraded obviously, on account of the lengthy procedure of this method (Fig. 2D). Thus, the Kit method is an optimized method to obtain high-quality total RNA from inner scales of *L. davidii* var*. unicolor* (Table 2; Fig. 2). In comparison with the TRIzol and CTAB methods, the modified TRIzol method could also extract high-quality total RNA from the inner scales.

### High quantity and integrity RNA was obtained through the modified TRIzol method from the middle and external scales

For middle and external scales of *L. davidii* var*. unicolor*, the A260/A230 ratios of RNA samples ranged from 0.29 to 1.39, from 1.85 to 1.99, and from 1.55 to 1.89 for the modified TRIzol method, the Kit method and the CTAB method, respectively (Table 3), indicating that the RNA samples extracted by the modified TRIzol and CTAB methods were contaminated by polyphenols and polysaccharides. There was no significant difference in A260/A280 ratios among these three methods, and the values were all higher than 2.0, demonstrating that all of the RNA samples were free of proteins and DNA contaminations. However, it is remarkable that when compared with the Kit and CTAB methods, the modified TRIzol method obtained the most enriching total RNA (ranged from 460.92 to 1581.90 ng μL^−1^) (Table 3).

**Table 3:** Average purity and yield of total RNA extracted from middle and external scales of *Lilium davidii* var. *unicolor* using different methods.

Parts	260/230			260/280			Concentration ( ng μL^−1^)		
	Modified TRIzol	Kit	CTAB	Modified TRIzol	Kit	CTAB	Modified TRIzol	Kit	CTAB
T-middle	0.48 ± 0.06c	1.96 ± 0.01a	1.64 ± 0.11b	2.26 ± 0.01a	2.23 ± 0.00a	2.16 ± 0.04b	1019.69 ± 22.38a	458.83 ± 3.81b	505.67 ± 44.09b
M-middle	0.29 ± 0.01c	1.93 ± 0.02a	1.55 ± 0.013b	2.25 ± 0.01a	2.20 ± 0.01a	2.11 ± 0.03b	748.71 ± 13.24a	414.52 ± 19.48b	344.76 ± 58.54b
B-middle	1.39 ± 0.42a	1.99 ± 0.01a	1.81 ± 0.15a	2.26 ± 0.01a	2.25 ± 0.00a	2.20 ± 0.04a	1581.90 ± 54.03a	688.25 ± 65.19b	448.49 ± 24.13c
T-external	0.45 ± 0.22b	1.85 ± 0.02a	1.89 ± 0.02a	2.18 ± 0.01b	2.19 ± 0.01b	2.25 ± 0.02a	460.92 ± 44.47a	374.31 ± 50.34a	180.32 ± 20.86b
M-external	0.58 ± 0.27b	1.86 ± 0.02a	1.57 ± 0.10a	2.19 ± 0.01a	2.20 ± 0.01a	2.16 ± 0.02a	519.83 ± 107.83a	339.20 ± 29.01ab	156.53 ± 17.00b
B-external	1.04 ± 0.24b	1.97 ± 0.01a	1.55 ± 0.2ab	2.21 ± 0.01ab	2.24 ± 0.00a	2.12 ± 0.06b	590.75 ± 85.80a	763.99 ± 16.73a	203.44 ± 10.93b

*T* The top part of the detected scales, *M* The middle part of the detected scales, *B* The basal part of the detected scales.

**Figure 3:**
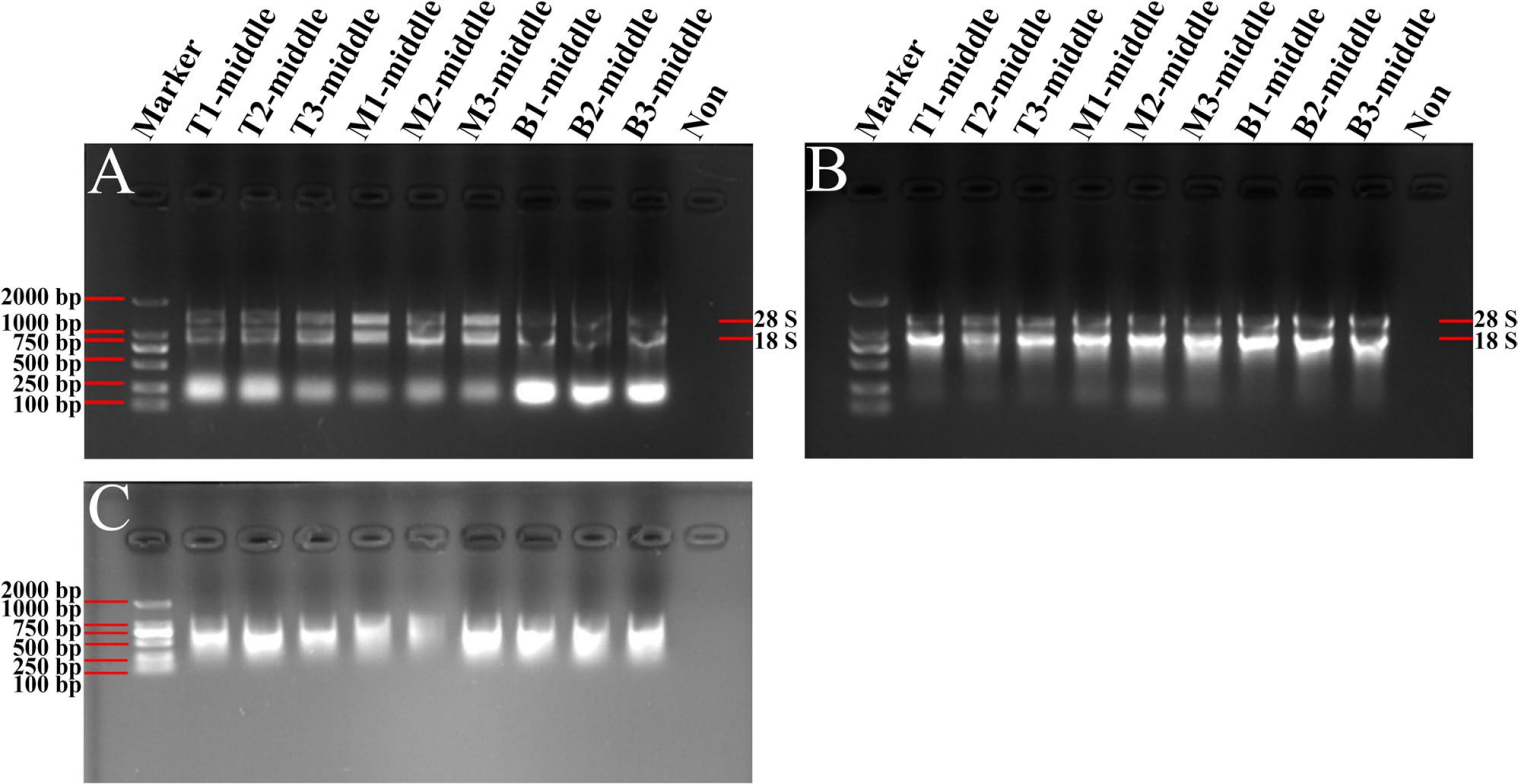
Gel images of total RNA from middle scales of *Lilium davidii* var. *unicolor* isolated using different protocols. The RNA samples extracted by the modified TRIzol method (**A**), Kit method (**B**) and CTAB method (**C**) were separated and analyzed in 1% agarose gels, respectively. Then the gels were visualized and exposed by Gel Imager System in different fields, and pictures were taken, respectively. The order of samples of each gel lane is as follows: Lane 1: DL2000 DNA marker; Lanes 2–4 are RNA isolated from top scales of *Lilium*; Lanes 5–7 are RNA isolated from middle scales of *Lilium*; Lanes 8–10 are RNA isolated from basal scales of *Lilium*; Lane 11: Loading buffer solution without RNA to serve as a control. 28S and 18S represent the location of 28S and 18S rRNA bands.

**Figure 4:**
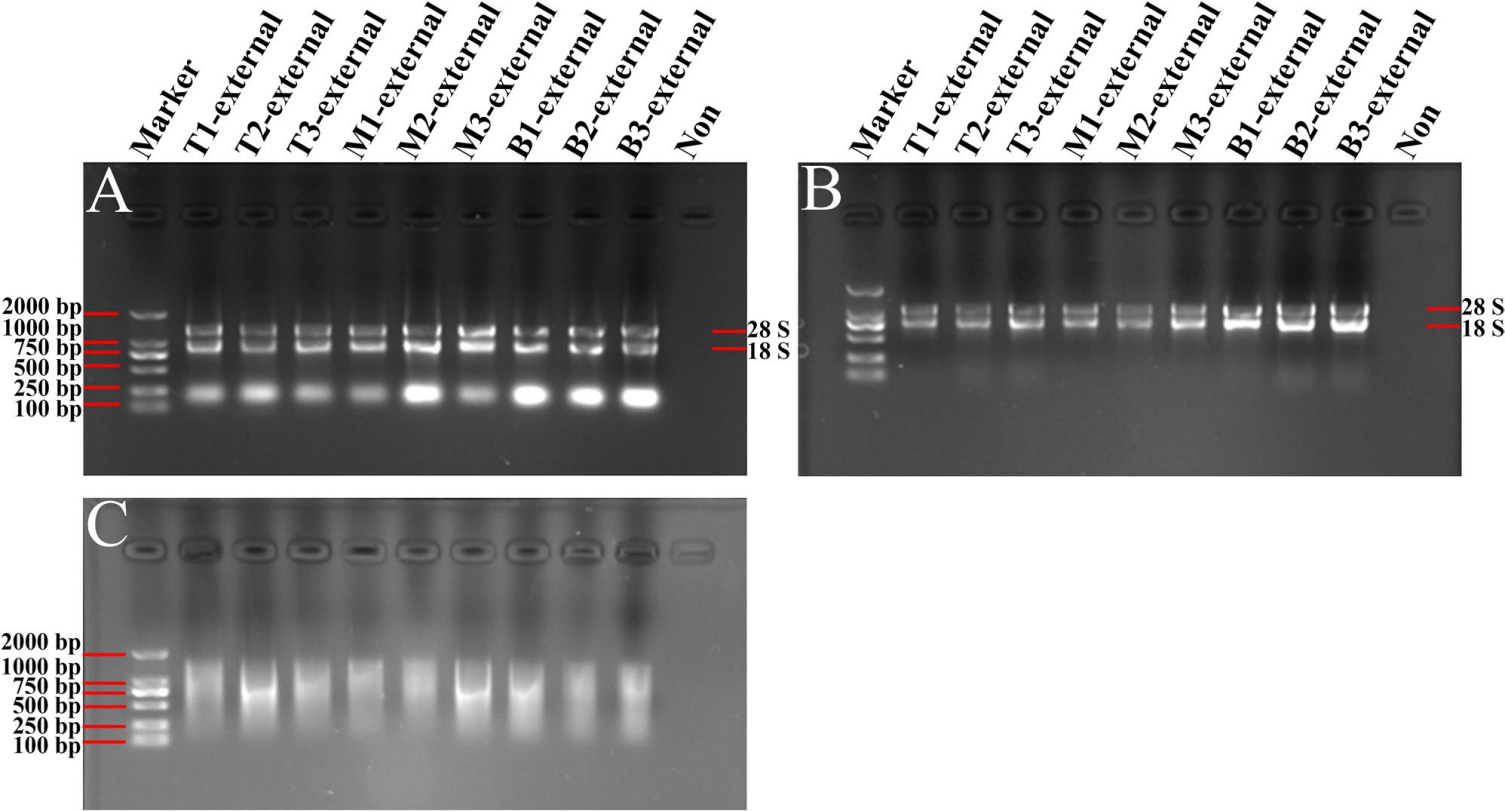
Gel images of total RNA from external scales of *Lilium davidii* var. *unicolor* isolated using different protocols. The RNA samples extracted by the modified TRIzol method (**A**), Kit method (**B**) and CTAB method (**C**) were separated and analyzed in 1% agarose gels, respectively. Then the gels were visualized and exposed by Gel Imager System in different fields, andpictures were taken, respectively. The order of samples of each gel lane is as follows: Lane 1: DL2000 DNA marker; Lanes 2–4 are RNA isolated from top scales of *Lilium*; Lanes 5–7 are RNA isolated from middle scales of *Lilium*; Lanes 8–10 are RNA isolated from basal scales of *Lilium*; Lane 11: Loading buffer solution without RNA to serve as a control. 28S and 18S represent the location of 28S and 18S rRNA bands.

Meanwhile, 1% agarose gel electrophoresis showed that the RNA bands were clear and complete from the modified TRIzol method and the Kit method (Figs. 3A,B, 4A,B). Nevertheless, the total RNA obtained by use of the CTAB method was degraded seriously (Figs. 3C, 4C). Taken together, total RNA concentrations obtained from the middle and external scales by using the modified TRIzol method were higher than the other methods, and the RNA bands were clear and intact (Table 3; Figs. 3, 4). What is noteworthy is that the RNA separated by all these methods from the basal part of *Lilium* scales showed higher purity and concentration than from other parts of the scales.

### The modified TRIzol method is preferable for obtaining RNA with high quantity from the inner scales of other edible bulbs

In order to evaluate whether the modified TRIzol method is effective in extracting RNA from other kinds of edible bulbs, the TRIzol and modified TRIzol methods were employed simultaneously to gain RNA samples from the inner scales of *Lilium lancifolium* Thunb. and *Lilium brownii* var. *viridulum* Baker*.* As for samples of *Lilium lancifolium* Thunb., all the A260/A230 ratios were below 0.5. The A260/A230 ratios of RNA samples from the TRIzol method were improved mildly compared with that from the modified TRIzol method (Table 4). The A260/A280 ratios of the RNA samples extracted from the top-inner scales were similar for the TRIzol and modified TRIzol methods (2.11 and 2.12, respectively), whereas the A260/A280 ratios of RNA samples from middle-inner scales and basal-inner scales for the modified TRIzol method (1.95 and 1.99, respectively) were significant higher than that for the TRIzol method (1.56 and 1.71, respectively). In addition, the RNA concentration of middle-inner scales from the modified TRIzol method (88.53 ng μL^−1^) was higher than that from the TRIzol method (53.11 ng μL^−1^), while the yields of RNA of top-inner scales and basal-inner scales from the modified TRIzol method were relatively low (Table 4). Similarly, the A260/A230 and A260/A280 ratios of RNA from inner scales of *Lilium brownii* var. *viridulum* Baker for the modified TRIzol method were relatively higher than that for the TRIzol method, and the RNA concentrations from different parts of the inner scales extracted by the modified TRIzol method (ranged from 45.17 to 87.79 ng μL^−1^) were significantly higher than that by the TRIzol method (ranged from 9.24 to 35.90 ng μL^−1^) (Table 4).

**Table 4:** Average purity and yield of total RNA extracted from inner scales of other *Lilium* species using the two TRIzol methods.

Parts	260/230		260/280		Concentration (ng μL^−1^)	
	TRIzol	Modified TRIzol	TRIzol	Modified TRIzol	TRIzol	Modified TRIzol
A’-T-inner	0.26 ± 0.03	0.47 ± 0.10	2.11 ± 0.01	2.12 ± 0.01	161.24 ± 0.34	81.88 ± 1.51**
A’-M-inner	0.37 ± 0.04	0.32 ± 0.01	1.56 ± 0.04	1.95 ± 0.05**	53.11 ± 0.19	88.53 ± 5.56**
A’-B- inner	0.39 ± 0.01	0.34 ± 0.00	1.71 ± 0.01	1.99 ± 0.02**	100.09 ± 0.50	96.72 ± 0.48**
B’-T-inner	0.25 ± 0.03	0.31 ± 0.00	1.81 ± 0.12	1.97 ± 0.01	9.24 ± 0.66	87.79 ± 1.74**
B’-M-inner	0.39 ± 0.02	0.30 ± 0.00**	2.00 ± 0.05	1.93 ± 0.01	19.85 ± 0.07	71.07 ± 1.84**
B’-B-inner	0.24 ± 0.00	0.28 ± 0.00*	1.87 ± 0.04	2.07 ± 0.06*	35.90 ± 0.62	45.17 ± 1.63**

A’: *Lilium lancifolium* Thunb.; B’: *Lilium brownii* var. *viridulum* Baker; T: The top part of the detected scales; M: The middle part of the detected scales; B: The basal part of the detected scales. Values with asterisks represent significant difference between the two TRIzol methods by using *T* test in SPSS 24.0 software (*P < 0.05 and **P < 0.01).

**Figure 5:**
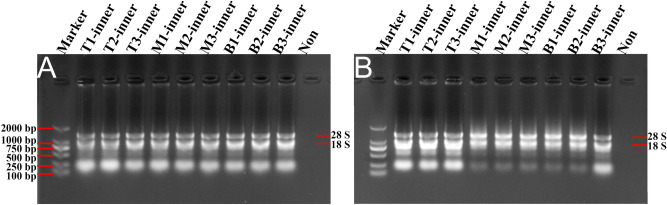
Gel images of total RNA from inner scales of *Lilium lancifolium* Thunb. and *Lilium brownii* var. *viridulum* Baker isolated using the modified TRIzol method. The RNA samples extracted by the modified TRIzol method were separated and analyzed in 1% agarose gels, respectively. Then the gels were visualized and exposed by Gel Imager System in different fields, and pictures were taken, respectively. The order of samples of each gel lane is as follows: (**A**) Lane 1: DL2000 DNA marker; Lanes 2–4 are RNA isolated from top scales of *Lilium lancifolium* Thunb.; Lanes 5–7 are RNA isolated from middle scales of *Lilium lancifolium* Thunb.; Lanes 8–10 are RNA isolated from basal scales of *Lilium lancifolium* Thunb.; Lane 11: Loading buffer solution without RNA to serve as a control; (**B**) Lane 1: DL2000 DNA marker; Lanes 2–4 are RNA isolated from top scales of *Lilium brownii* var. *viridulum* Baker; Lanes 5–7 are RNA isolated from middle scales of *Lilium brownii* var. *viridulum* Baker; Lanes 8–10 are RNA isolated from basal scales of *Lilium brownii* var. *viridulum* Baker; Lane 11: Loading buffer solution without RNA to serve as a control*.* 28S and 18S represent the location of 28S and 18S rRNA bands.

**Figure 6:**
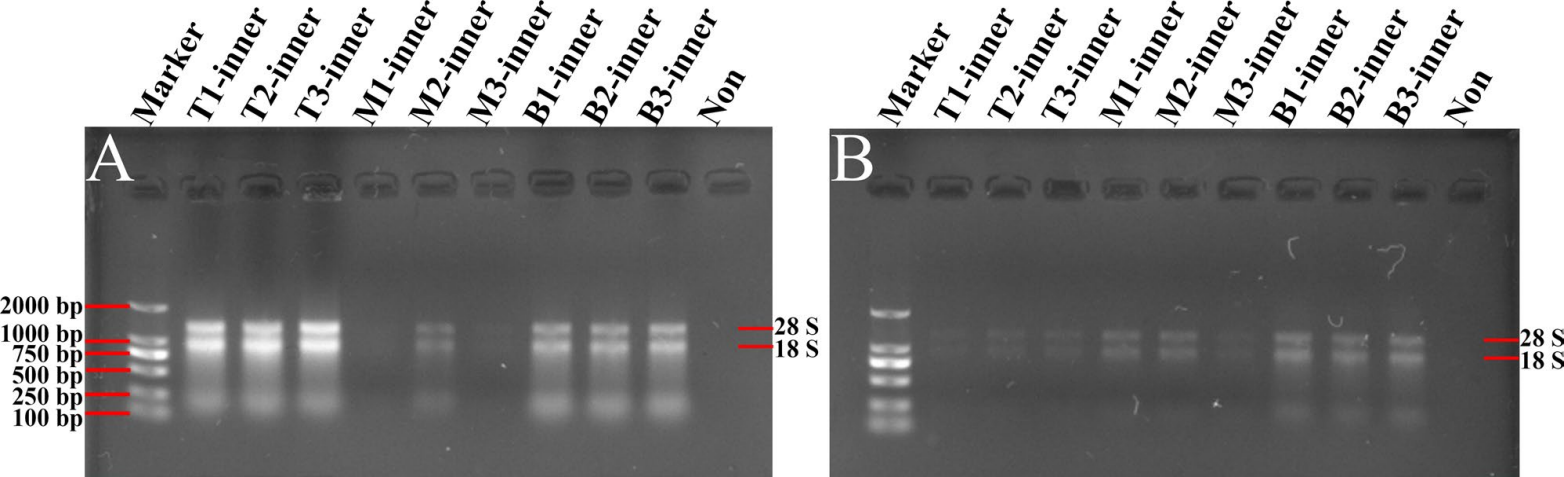
Gel images of total RNA from inner scales of *Lilium lancifolium* Thunb. and *Lilium brownii* var. *viridulum* Baker isolated using TRIzol method. The RNA samples extracted by TRIzol method were separated and analyzed in 1% agarose gels, respectively. Then the gels were visualized and exposed by Gel Imager System in different fields, and pictures were taken, respectively. The order of samples of each gel lane is as follows: (**A**) Lane 1: DL2000 DNA marker; Lanes 2–4 are RNA isolated from top scales of *Lilium lancifolium* Thunb.; Lanes 5–7 are RNA isolated from middle scales of *Lilium lancifolium* Thunb.; Lanes 8–10 are RNA isolated from basal scales of *Lilium lancifolium* Thunb.; Lane 11: Loading buffer solution without RNA to serve as a control; (**B**) Lane 1: DL2000 DNA marker; Lanes 2–4 are RNA isolated from top scales of *Lilium brownii* var. *viridulum* Baker; Lanes 5–7 are RNA isolated from middle scales of *Lilium brownii* var. *viridulum* Baker; Lanes 8–10 are RNA isolated from basal scales of *Lilium brownii* var. *viridulum* Baker; Lane 11: Loading buffer solution without RNA to serve as a control*.* 28S and 18S represent the location of 28S and 18S rRNA bands.

Further, the RNA bands from these two edible bulbs were clear and complete gained from the modified TRIzol method in 1% agarose gel electrophoresis analysis (Fig. 5), while RNA bands extracted from the TRIzol method were weak or absent (Fig. 6). In all, the above results suggest that more sufficient total RNA can be obtained by applying the modified TRIzol method in edible bulbs of different *Lilium* species, though the contamination of polysaccharides, polyphenols and proteins were similar in RNA samples extracted from the two TRIzol methods.

## Discussion

*L. davidii* var. *unicolor* has been regarded as both the vegetable and the herbal medicine for several centuries in China because its bulb could provide polysaccharides and phenolic compounds to treat bronchitis, pneumonia and provide nutritions^22,23^. Amounts of polyphenols and polysaccharides are accumulated during the bulb formation and development stages of *Lilium*. Hence, extracting high-quality RNA samples from *Lilium* bulb is a tough process as polysaccharides and polyphenols tend to co-precipitate with RNA^24,25^. The metabolite composition of plant tissues varies greatly, so different approaches are required to obtain high-quality RNA^16,26^. Several RNA extraction methods have been reported in seed or storage root of pistachio^14^, cassava^16^, *Arabidopsis*^19^, sunflower^27^, Jerusalem artichoke^24^ and sugar beet^28^, including the TRIzol method. Over the past decade, TRIzol, Kit and CTAB methods were used to isolate total RNA from leaf, root and bulb of Lanzhou lily^19–21,29^. However, which method is more suitable of extracting total RNA from different tissues in this kind of *Lilium* is still needed to be explored. In this study, several methods were researched to obtain high-quality and integrated total RNA in an easy and low-cost condition from different tissues in *Lilium*, and the optimal RNA isolation method was selected and optimized.

In the present study, the TRIzol method was introduced to isolate total RNA from root, stem, leaf and inner scales of Lanzhou lily, but resulted in low yield and divergent degradation (Figs. 1, 2). Meanwhile, we initially tried to extract RNA samples from different tissues of *Lilium* by applying the CTAB and Kit approaches, but the results remained unsatisfactory (Fig. 1). However, compared with the CTAB and Kit methods, the modified TRIzol method could extract high-quality RNA from different tissues of *Lilium* (Table 1). A previous study reported an improved TRIzol method to obtain total RNA from the bulb of Lanzhou lily, but it was failed to remove polysaccharides in *Lilium*^17^. Another version of modified TRIzol method could effectively eliminate the above contaminations by adding sodium acetate solution, DTT and β-mercaptoethanol reagents in the extracted procedure^19^, suggesting the potential role of TRIzol method in extracting RNA from Lanzhou lily. During the operation of the modified TRIzol method in this study, after the RNA was precipitated by isopropanol, changing the extraction environment to 4 °C, using the RNA purification adsorption column and washing the RNA pellet with ethanol repeatedly are critical procedures for obtaining high quality and purity RNA samples. Furthermore, large amount of TRIzol reagents should be used to effectively dissolve tissue samples and gain more supernatant, resulting in an increased quantity of total RNA. Among these testified methods, when using the modified TRIzol method, the highest RNA concentration was obtained from root, stem, leaf (Table 1) and different parts of scales (Tables 2, 3) in *Lilium*, with the A260/A280 ratios ranging from 1.97 to 2.27. Although the A260/A230 ratios for different tissues were still low, the 28S and 18S rRNA bands could be clearly observed, confirming the integrity of the RNA samples isolated by the modified TRIzol method (Figs. 1, 2, 3, 4). These results were further certified by comparing the modified TRIzol method with the TRIzol method in other edible bulb samples (*Lilium lancifolium* Thunb. and *Lilium brownii* var. *viridulum* Baker) (Figs. 5, 6). The relatively lower RNA concentrations in these edible bulbs in comparison with Lanzhou lily may be caused by the long-term storage and transportation, while bulbs of Lanzhou lily were collected locally. In addition, for different scales of *Lilium*, it was easier to obtain high yield and quality total RNA from the basal part of the scales (Tables 2, 3). This might because that the basal part have the higher transcriptional activity and less content of polysaccharides and other secondary metabolites than other parts of the scales.

Previous studies have reported that CTAB method could extract high purity and yield of total RNA from different species which contained high content of polyphenols and polysaccharides^30,31^. Also, the CTAB method could obtain higher quality and quantity total RNA from leaf, root and bulb of Lanzhou lily^17,29^. In this study, while isolating RNA samples with the CTAB method from root, stem and leaf, one of the main obstacles is the existence of DNA contamination (Fig. 1). To further eliminate the interference of DNA from RNA samples, we added the RNA-free DNase I digestion step in the CTAB method for extracting RNA from different lily scales. In the scales of *Lilium*, as shown in Tables 2 and 3, the A260/A230 ratios of RNA samples isolated by the CTAB method were lower than that by the Kit method, but higher than that by the modified TRIzol method. When using the CTAB method, the A260/A280 ratios of the separated RNA samples were above 2.1 except for RNA samples of stem and leaf. The above results indicate that the CTAB method could effectively remove proteins, but couldn’t avoid the contamination of polysaccharides and polyphenols in the isolated total RNA samples. Unexpectedly, the RNA bands in 1% agarose gel electrophoresis gained from the CTAB method were incomplete and diffused (Figs. 2, 3, 4), indicating that the RNA samples from the scales isolated by the CTAB method were degraded, though the contamination of DNA was obviously reduced. So we speculated that this might be due to the long time spent in the process of RNA extraction by the CTAB method, which led to the degradation of RNA. As reported before, the improved CTAB method is suitable for extracting high purity, intact and high yield RNA than the improved TRIzol method in *Lilium*^30^. However, compared with the modified TRIzol method, the CTAB method here obtained lower concentration and lower yield RNA in Lanzhou lily (Tables 1, 2, 3). The yield of RNA extracted by the CTAB method is reduced due to the high affinity of CTAB with nucleic acids and other biopolymers^32^. In addition, the process of the CTAB method is more complicated and more preparations are needed before the experiment compared with the modified TRIzol method, as the modified TRIzol method is easier to be conducted, and no solution except for 75% ethanol is needed to be prepared before. Thus, the modified TRIzol method may be more suitable than the CTAB method for acquiring total RNA samples from *L. davidii* var. *unicolor*.

Compared with the CTAB method, the Kit method resulted in higher yield and purity RNA from bulb scales that contain high levels of polysaccharides and polyphenols. Previous studies reported that Kit method could be used to isolate RNA from bulb of Lanzhou lily^20,21^. However, the Kit protocol is not suitable for extracting total RNA from root, stem and leaf of *Lilium* in the current study (Table 1). Meanwhile, the Kit protocol is the most expensive method among these methods, considering the extra amount of reagents used for extracting high quality and purity RNA from *L. davidii* var. *unicolor*. In our results, the modified TRIzol method could obtain more enriching and integrated RNA samples, though the RNA contains a contamination of polysaccharides and polyphenols. Together with the fact that the Kit method is overspent, and there is a complicated and interminable process within the CTAB method, we suggested that the modified TRIzol protocol is the efficient, simple and economical method to acquire high-quality and integrity total RNA from different tissues in *Lilium*.

## Conclusion

TRIzol method, modified TRIzol method, CTAB method and Kit method were used to extract RNA from tissues in *L. davidii* var. *unicolor*, and the highest quality and yield total RNA were obtained from the modified TRIzol protocol. In addition, the intense and intact RNA bands in 1% agarose gel electrophoresis of the modified TRIzol method showed a distinct advantage among these methods. Furthermore, this method is an easy, efficient, and low-cost method for total RNA isolation from *Lilium*. In all, the modified TRIzol method is sufficient to gain eligible RNA in *Lilium* to support further molecular experiments.

## Materials and methods

### Plant materials

**Figure 7:**
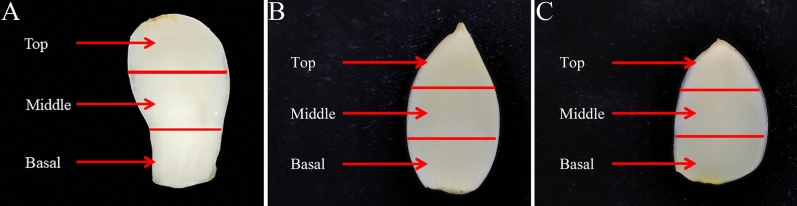
Different layers of bulb scales from different edible lily species used in the study. Different layers of bulb scales from different edible lily species were sorted into three equal parts, named as top part, middle part and basal part. (**A**) Different layers of bulb scales from *Lilium davidii* var. *unicolor*; (**B**) Different layers of bulb scales from *Lilium lancifolium* Thunb.; (**C**) Different layers of bulb scales from *Lilium brownii* var. *viridulum* Baker.

Lily plants ‘*L. davidii* var. *unicolor*’ were cultivated in the greenhouse (the average temperature was 18.5 °C and the average degree of humidity was 79.4%) of Gansu Agricultural University, Lanzhou, Gansu Province, China (36.10384 N, 103.7189 E). Firstly, fresh plants were collected and washed with deionized sterile water and dissected leaf, stem, root and bulb separately. Secondly, scales were removed carefully from mother bulb and sorted into three groups: external scales (1–3 layers), middle scales (4–6 layers) and inner scales (7–9 layers). Then different scales were sorted into three equal parts, named as top part, middle part and basal part (Fig. 7A). Thus, the different tissues from *Lilium* were named successively as top-inner scales, middle-inner scales, basal-inner scales, top-middle scales, middle-middle scales, basal-middle scales, top-external scales, middle-external scales and basal-external scales. Lily bulbs of *Lilium lancifolium* Thunb. were produced in Enshi, Hubei Province, China (110.09242 N, 30.2223 E) and harvested after the aerial part of the plants withered in October 2021. Lily bulbs of *Lilium brownii* var. *viridulum* Baker were produced in Longhui, Hunan Province, China (111.03249 N, 27.1140 E) and harvested after the aboveground part of the plants withered in August 2021. Different layers of the tested inner scales of these two edible bulbs were collected by the same method used in *L. davidii* var. *unicolor* (Fig. 7B,C). The above samples were immediately frozen in liquid nitrogen and then preserved at − 80 °C. All experiments conducted in this study, including the collection of plant materials, were in compliance with relevant institutional, national, and international guidelines and legislations.

### Reagents and solutions

Plastic materials (tubes and pipette tips), mortars and pestles used in the current paper were soaked overnight in 0.01% DEPC-treated distilled water and then autoclaved at 121 °C for 1 h. In our methods, TRIzol® reagent (Invitrogen, USA) and TaKaRa MiniBEST Plant RNA Extraction Kit (TaKaRa, Japan) were applied. Buffers and solutions were prepared as follows. CTAB extraction buffer: 2% CTAB (w/v); 2% soluble polyvinylpyrrolidone (PVP, w/v); 100 mM Tris–HCl (pH 8.0); 2 M NaCl; 25 mM ethylenediamine tetraacetic acid (EDTA); 0.5 g L^−1^ spermidine and 3% β-mercaptoethanol (added to pre-heated autoclaved buffer prior to extraction). Other reagents included chloroform-isoamylalcohol (C: I) at a ratio of 24:1, 8 M LiCl and 75% ethanol.

### RNA extraction protocols

#### Method 1: Modified TRIzol method

TRIzol-based RNA extraction was done according to Ahmad et al. (2017), and the modified TRIzol method was done according to this TRIzol method with some modifications^33^. In order to obtain higher concentration of total RNA, RNA purification adsorption column were used after Steps 4. Our modified procedure is described as follows: (1) About 300 mg of each tissue samples was ground into a fine powder using liquid nitrogen and transferred into a 1.5 mL centrifuge tubes. Then 1 mL of TRIzol reagent was added, and mixed by a micropipettor. Incubated the samples for 10 min at 4 °C, and centrifuged at 12,000 g for 5 min at 4 °C. (2) The supernatant was transferred to a new 1.5 mL Eppendorf tube. 200 µL of cold chloroform was added and the mixture was hand-shaken and then incubated at room temperature for 5 min. Then it was centrifuged at 12,000 g for 15 min at 4 °C. (3) The supernatant (~ 700 μL) was transferred to a new 1.5 mL chilled Eppendorf tube and then an equal volume of chilled isopropanol was added and mixed by inverting the tube for 6–10 times. The final mixture was incubated at − 20 °C overnight to precipitate RNA. (4) The obtained RNA pellet from two repeats above was added to one RNA purification adsorption column (700 μL for each time) followed by a centrifugation of 15 min at 12,000 g at 4 °C to obtain a visible precipitate, and the supernatant was discarded. (5) 700 µL pre-cooled 75% ethanol were added and mixed by inverting the tube for 4–6 times, and centrifuged at 12,000 g for 1 min at 4 °C, then the supernatant were discarded. The ethanol washing step was repeated twice, then centrifuged at 12,000 g for 2 min at 4 °C and the redundant ethanol was discarded. (6) After centrifugation, the tubes were carefully transferred to the ice, and the washed RNA pellet was air-dried for 10 min in super clean bench. (7) 40 μL RNase free H_2_O was added onto the membrane of the RNA purification adsorption column to dissolve RNA. RNA samples were incubated at 4 °C for 3 min, and then centrifuged at 12,000 g for 2 min at 4 °C to elute RNA. To obtain high concentration of RNA, the first eluent was added back into the RNA purification adsorption column, incubated and centrifuged again as above.

#### Method 2: CTAB method

CTAB method was constructed according to White et al. (2008) with some modifications^10^, the RNase-free DNase I and RNA purification adsorption column were used in Steps 10 and 11, respectively. Our modified procedure is described as follows: (1) The extraction buffer was preheated in a 50 mL Eppendorf tube (with 3% β-mercaptoethanol added) and bathed at 65 °C for 30 min. (2) About 300 mg of each sample was grounded into a fine powder by using liquid nitrogen, then the sample was transferred to 1.5 mL chilled Eppendorf tube and 1.2 mL pre-heated extraction buffer was added. (3) The tube was vortexed immediately and then incubated at 65 °C in water bath for at least 30 min with vortexing once every 5 min. (4) The tube was centrifuged at 12,000 g for 10 min at 4 °C and the supernatant was transferred to a new 1.5 mL Eppendorf tube. (5) An equal volume (650–850 µL) of C: I (24:1) was added to the tube, with vortexing for 30 s and then centrifuged at 12,000 g for 15 min at 4 °C. (6) The aqueous phase was transferred to a new 1.5 mL Eppendorf tube without disruption of the white interphase. (7) Repeated C: I abstraction as in the previous steps (steps 5 and 6). (8) 1/3 volumes of 8 M pre-cooled LiCl was added to the supernatant in 1.5 mL Eppendorf tube and was mixed upside down, then the mixture was stored at 4 °C overnight for nucleic acid precipitation. (9) The obtained RNA pellet from two repeats above was combined and added to one RNA purification adsorption column (700 μL for each time) followed by a centrifugation of 15 min at 12, 000 g at 4 °C, then discarded the supernatant. (10) 5 µL 10 × DNase I Buffer, 4 µL Recombinant DNase I (TaKaRa, Tokyo, Japan, RNase free, 5 U µL^−1^) and 41 µL RNase free H_2_O were pipetted onto the membrane of the RNA purification adsorption column, followed by an incubation of 15 min at room temperature (25 °C) to eliminate possible genomic DNA contamination. (11) The RNA was washed, air-dried and eluted as described in steps 5–7 of the modified TRIzol protocol.

#### Method 3: TaKaRa MiniBEST Plant RNA Extraction Kit

Plant RNA Extraction Kit, which contained two types of cracking buffer named PE and RL, was used according to the manufacturer’s instructions (Cat. No. 9769). The method was modified when using RNA purification adsorption column to collecting RNA. In order to ensure high quality total RNA, the same volume of the treatment mixture were collected to the same adsorption column as in method 1 and 2. Importantly, all of the operating steps were performed at 4 °C to prevent RNA degradation during the experiment process.

### RNA assessment

The A230/260 and A260/280 ratios were measured by a Nano drop spectrophotometer to determine the purity of the extracted total RNA. The concentration of the RNA sample was detected simultaneously by the spectrophotometer to determine the RNA yield. RNA integrity was evaluated by the clarity of ribosomal RNA bands in 1% agarose gel electrophoresis^32^. The gels were stained with ethidium bromide (0.5 µg mL^−1^) and then visualized and documented by a Gel Imager System (GE, USA).

## Supplementary Information

Supplementary Information.

## Data Availability

All data generated or analyzed during this study are included in this article.
