# Supplementary material for: An optimized method to obtain high-quality RNA from different tissues in Lilium davidii var. unicolor
Source: Sci Rep. 2022 Feb 18;12:2825. doi: 10.1038/s41598-022-06810-7 (PMC8857280; doi:10.1038/s41598-022-06810-7)
Supplement: Supplementary file 1 — Supplementary Information. [file 41598_2022_6810_MOESM1_ESM.docx]

**An optimized method to obtain high-quality RNA from different tissues in *Lilium*** ***davidii* var. *unicolor***

Chunlei Wang^1^, Xuemei Hou^1^, Nana Qi^1^, Changxia Li^1^, Yanyan Luo^1^, Dongliang Hu^1^, Yihua Li^1^, and Weibiao Liao^1, *^

**Supplementary Files**


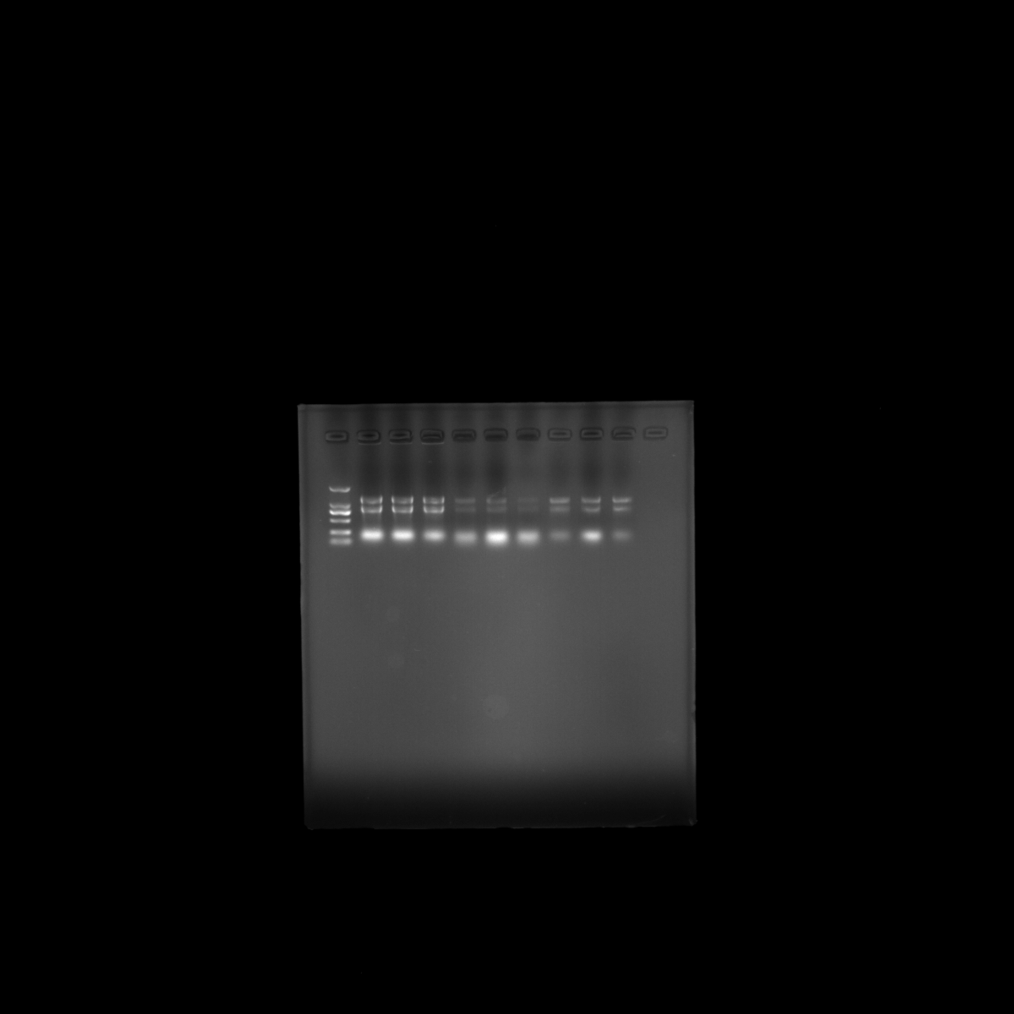


TRIzol


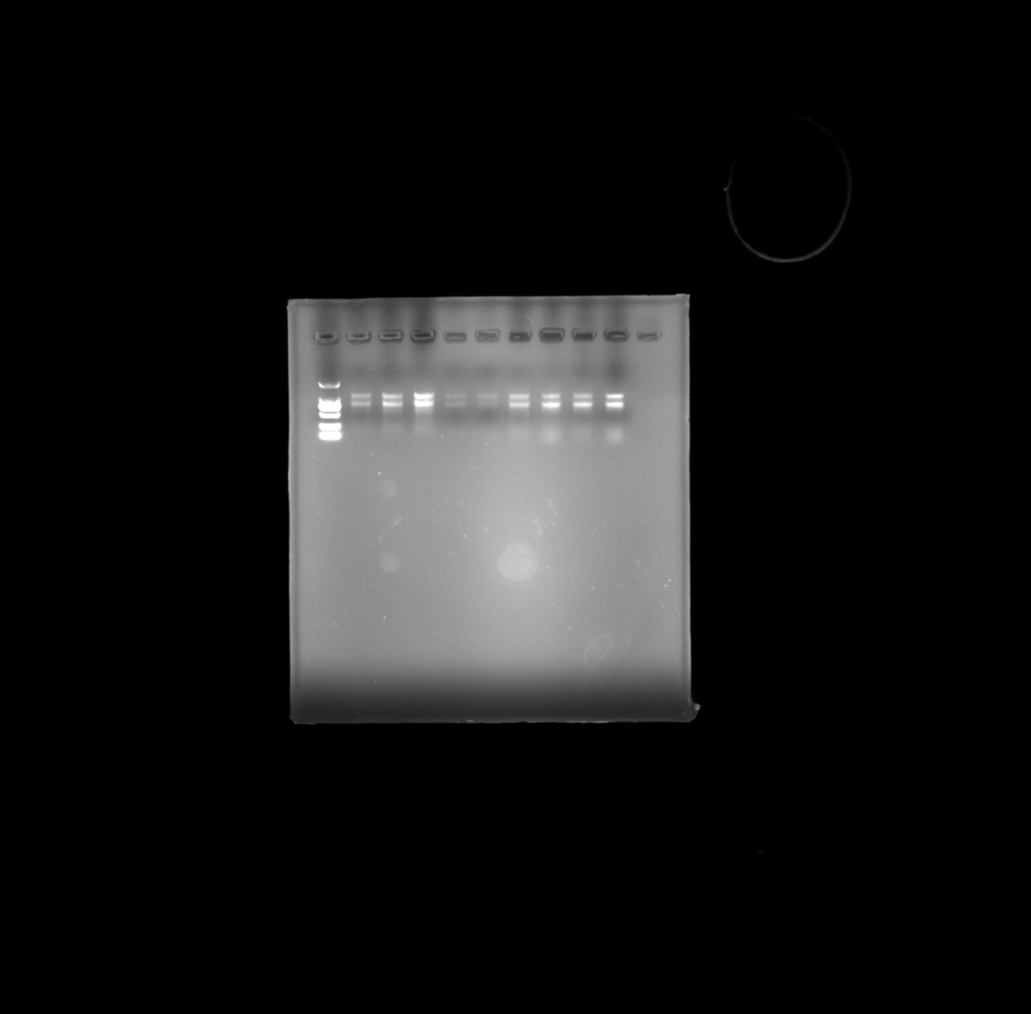


Kit


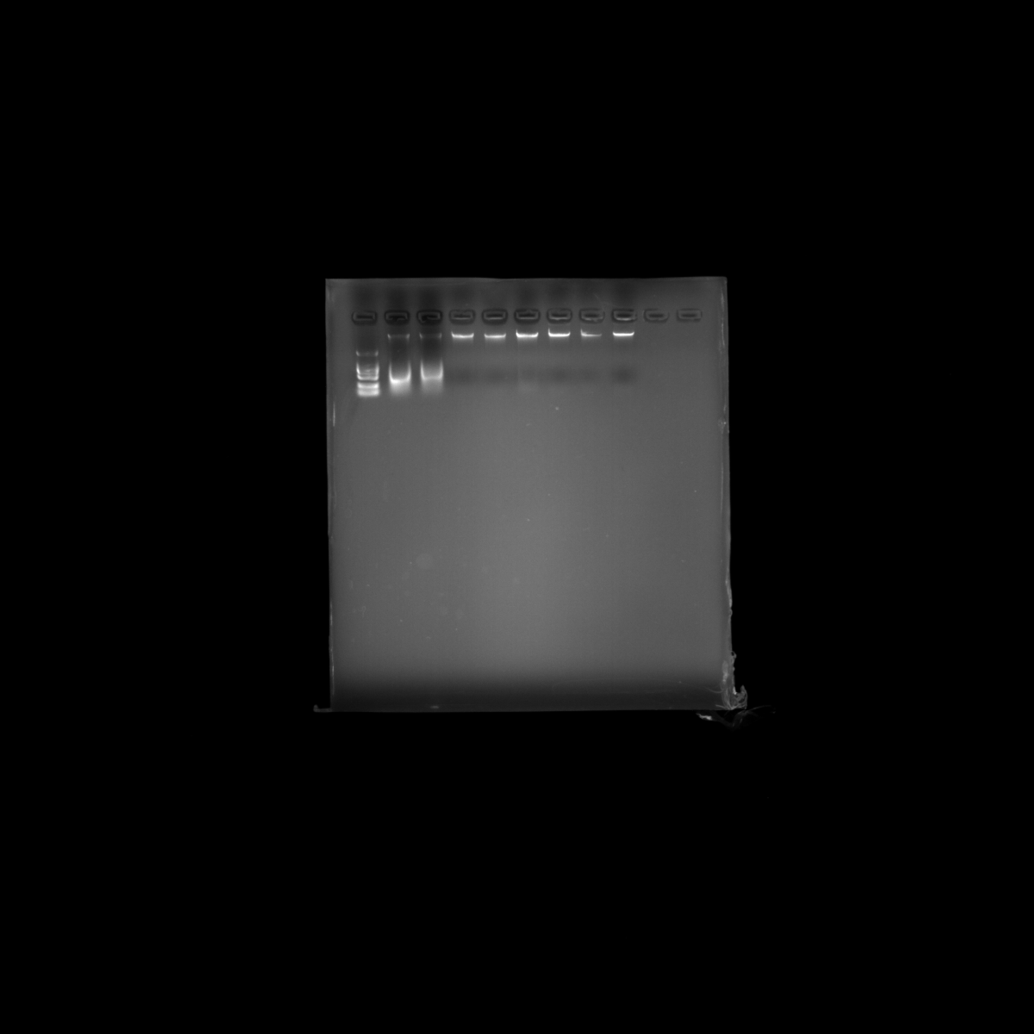


CTAB

**Supplementary Figure 1. The original full-length gels used to display total RNAs extracted from root, stem and leaf tissues of *Lilium davidii* var. *unicolor* by modified TRIzol, Kit and CTAB methods, respectively.** The order of samples in each gel lane is as follows: Lane 1: DL2000 DNA marker; Lanes 2-4 are RNA isolated from root of *Lilium*; Lanes 5-7 are RNA isolated from stem of *Lilium*; Lanes 8-10 are RNA isolated from leaf of *Lilium*; Lane 11: Loading buffer solution without RNA to serve as a control.


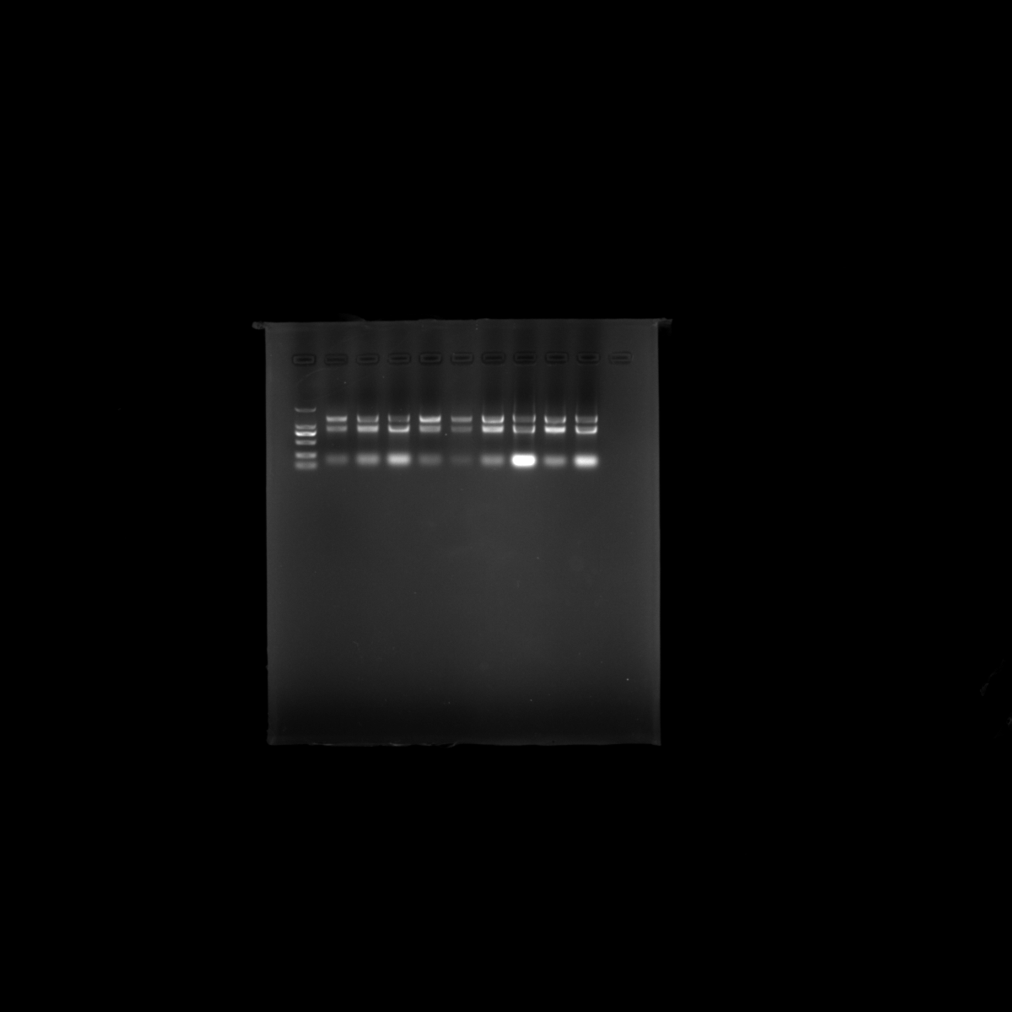


TRIzol


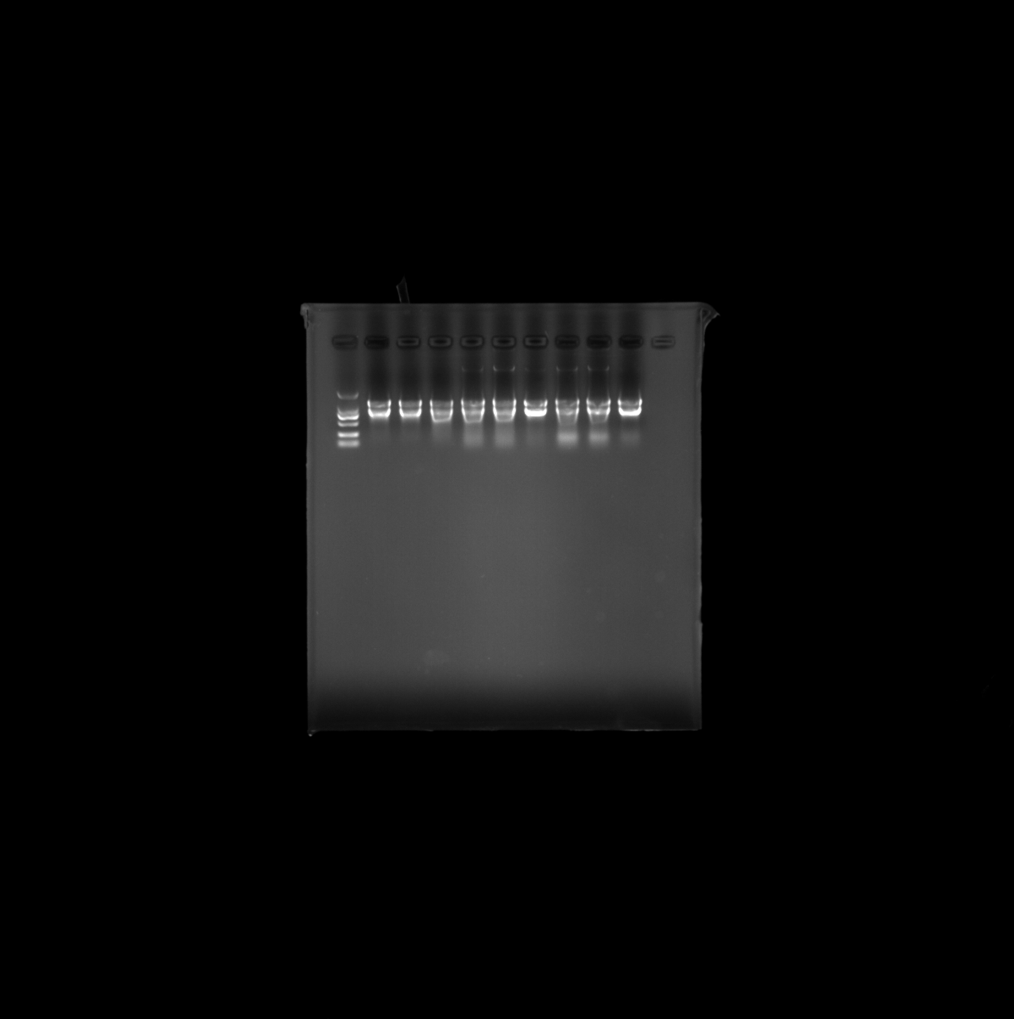


Kit


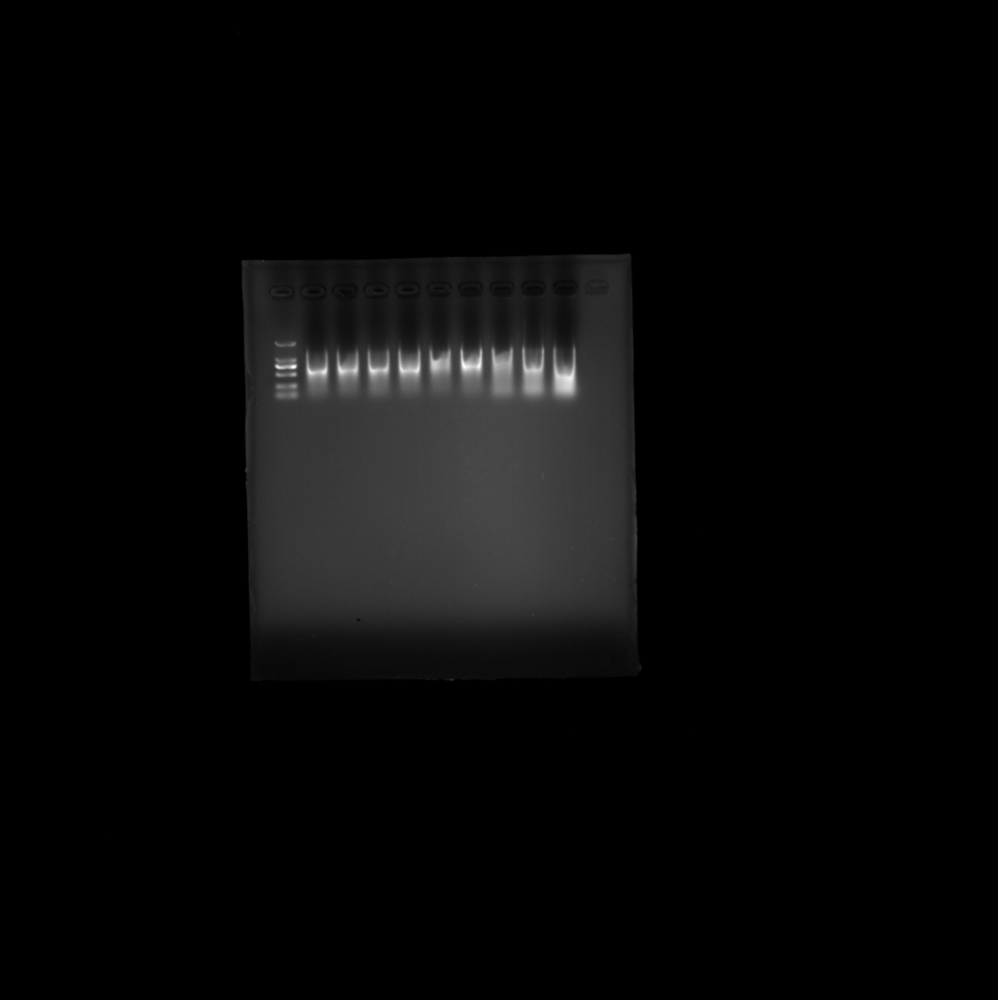


CTAB

**Supplementary Figure 2. The original full-length gels used to display total RNAs isolated from inner scales of *Lilium davidii* var. *unicolor* by using modified TRIzol, Kit and CTAB methods, respectively**. The order of samples in each gel lane is as follows: Lane 1: DL2000 DNA marker; Lanes 2-4 are RNA isolated from top scales of *Lilium*; Lanes 5-7 are RNA isolated from middle scales of *Lilium*; Lanes 8-10 are RNA isolated from basal scales of *Lilium*; Lane 11: Loading buffer solution without RNA to serve as a control.


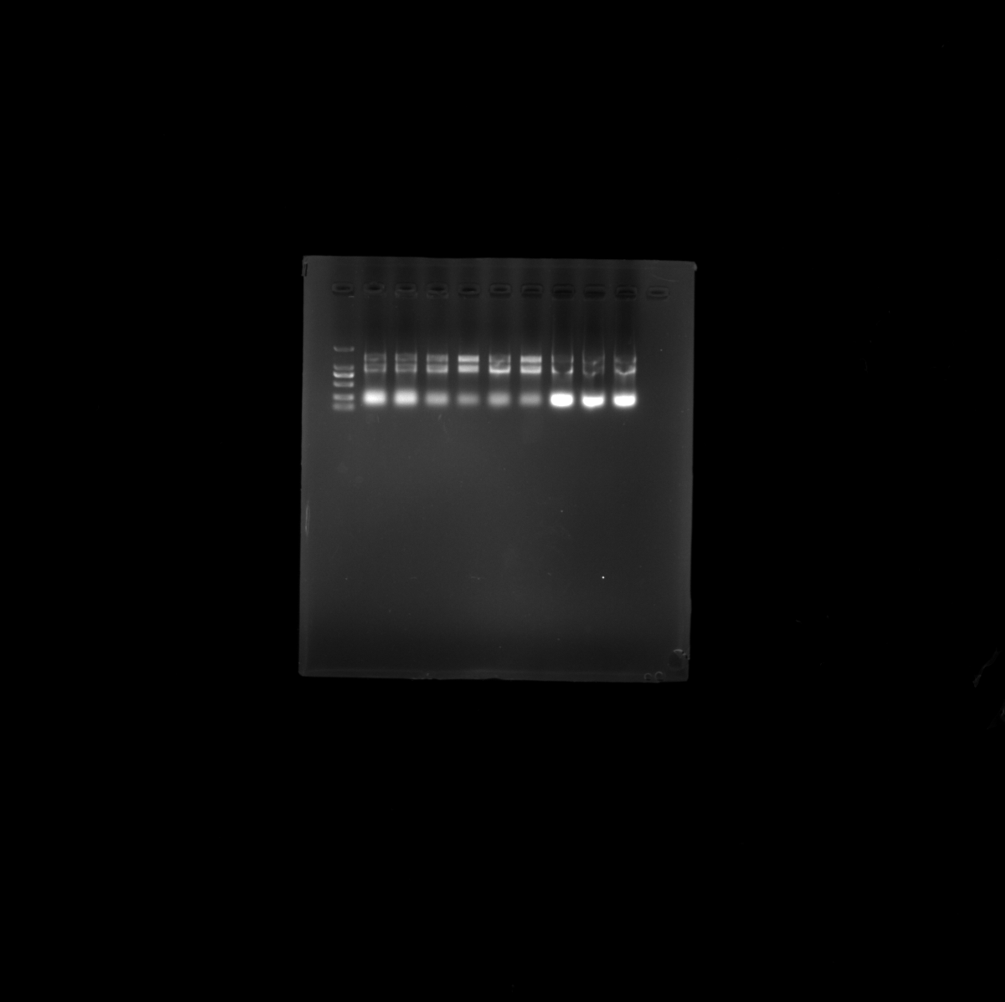


TRIzol


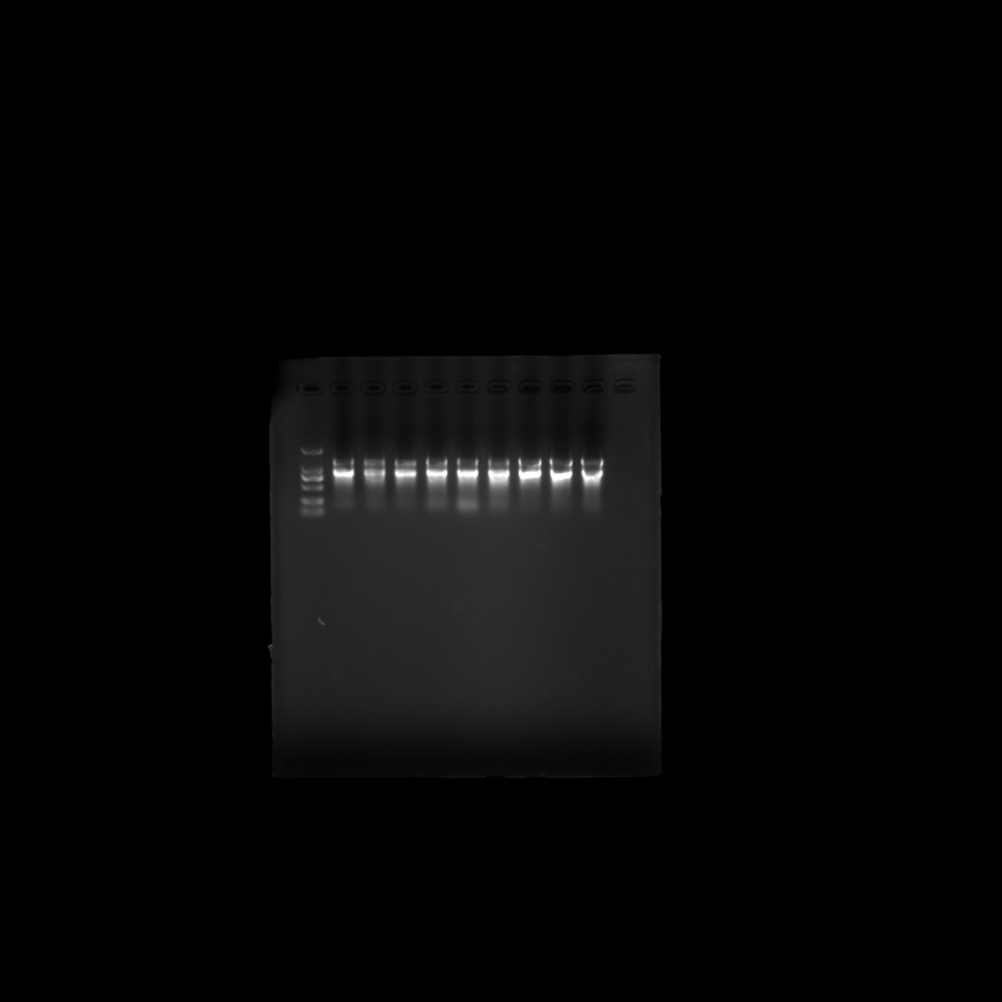


Kit


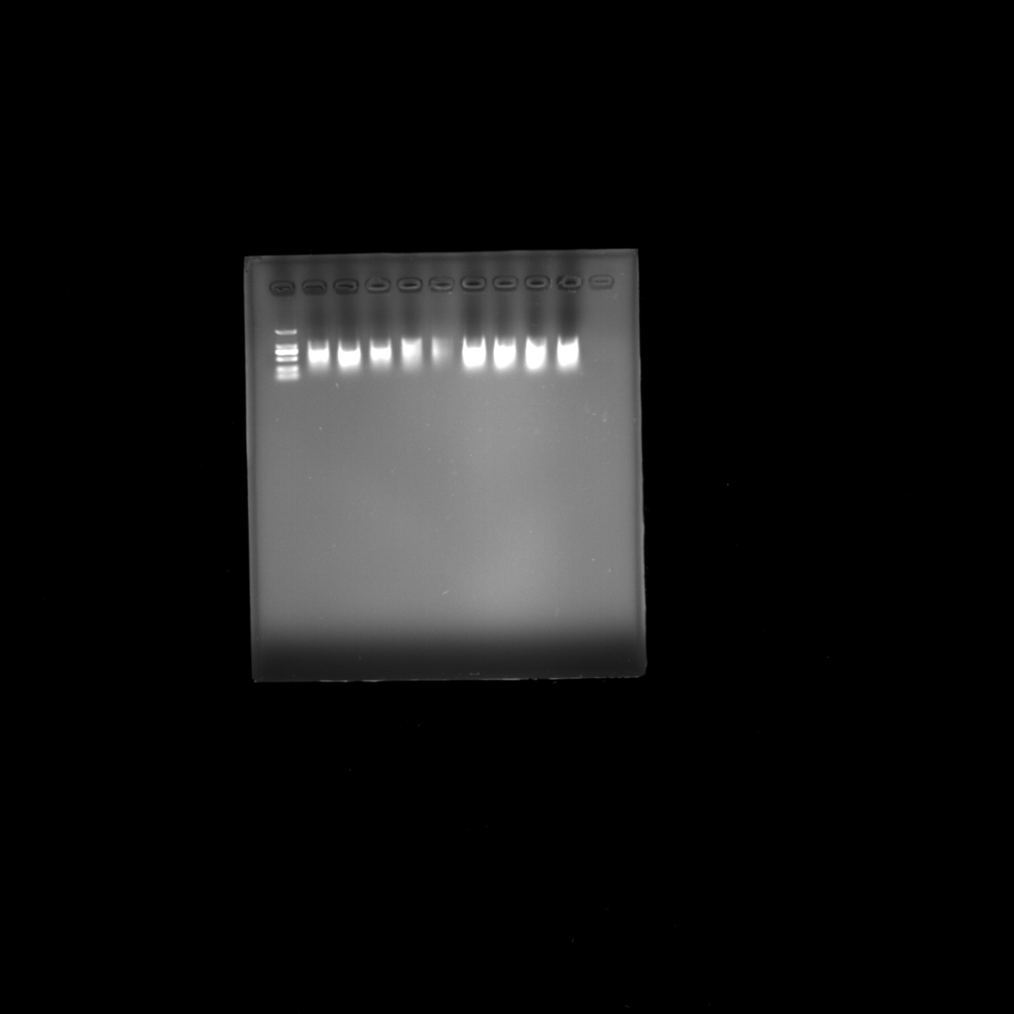


CTAB

**Supplementary Figure 3. The original full-length gels used to display total RNAs in middle scales of *Lilium davidii* var. *unicolor* isolated by modified TRIzol, Kit and CTAB methods, respectively**. The order of samples in each gel lane is as follows: Lane 1: DL2000 DNA marker; Lanes 2-4 are RNA isolated from top scales of *Lilium*; Lanes 5-7 are RNA isolated from middle scales of *Lilium*; Lanes 8-10 are RNA isolated from basal scales of *Lilium*; Lane 11: Loading buffer solution without RNA to serve as a control.

**Supplementary Figure 4. The original full-length gels used to display total RNAs in external scales of *Lilium davidii* var. *unicolor* isolated by using modified TRIzol, Kit and CTAB methods, respectively**. The order of samples in each gel lane is as follows: Lane 1: DL2000 DNA marker; Lanes 2-4 are RNA isolated from top scales of *Lilium*; Lanes 5-7 are RNA isolated from middle scales of *Lilium*; Lanes 8-10 are RNA isolated from basal scales of *Lilium*; Lane 11: Loading buffer solution without RNA to serve as a control.


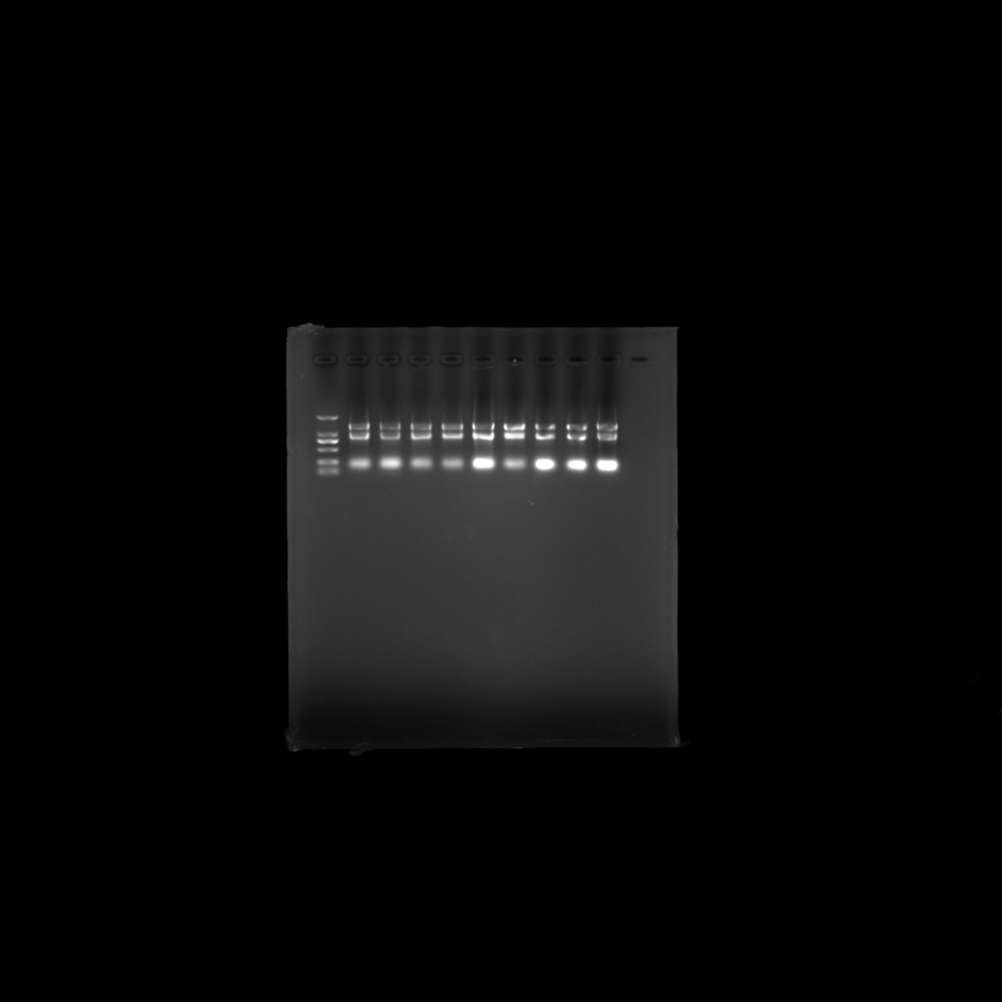


TRIzol


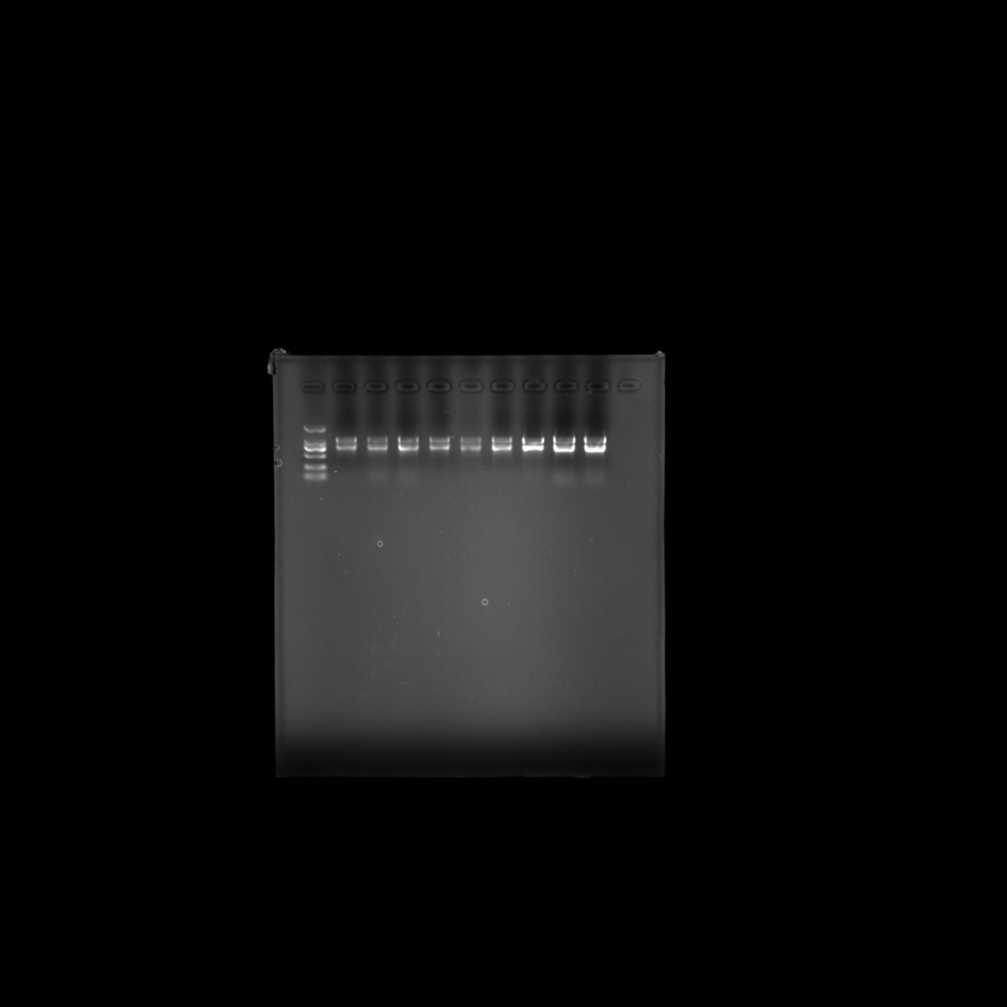


Kit

CTAB


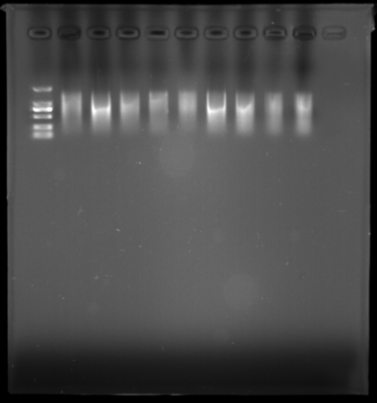


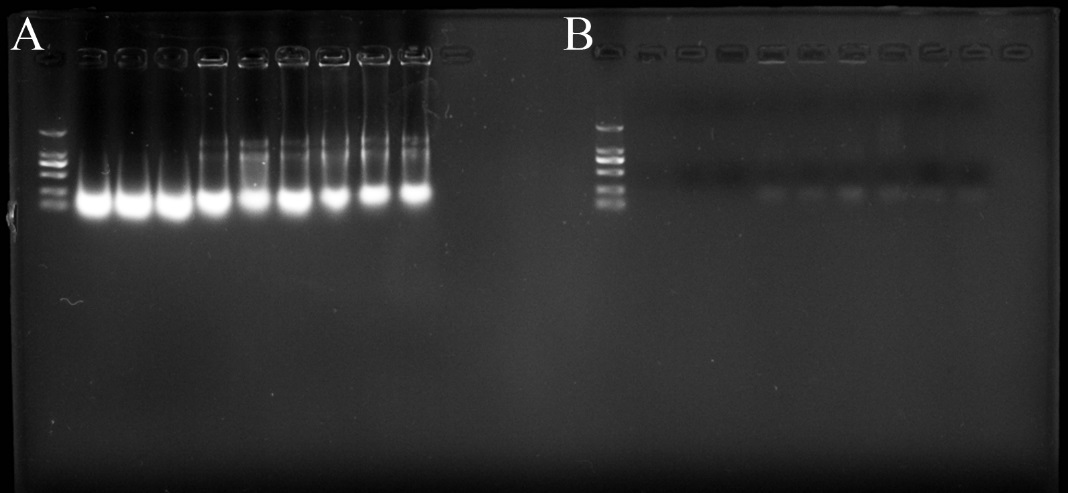


**Supplementary Figure 5. The original full-length gels used to display total RNAs in root, stem, leaf tissues and inner scales of *Lilium davidii* var. *unicolor* isolated by using TRIzol method**. The order of samplesin each gel lane is as follows: (A) Lane 1: DL2000 DNA marker; Lanes 2-4 are RNA isolated from root of *Lilium*; Lanes 5-7 are RNA isolated from stem of *Lilium*; Lanes 8-10 are RNA isolated from leaf of *Lilium*.; (B) Lane 1: DL2000 DNA marker; Lanes 2-4 are RNA isolated from top scales of *Lilium*; Lanes 5-7 are RNA isolated from middle scales of *Lilium*; Lanes 8-10 are RNA isolated from basal scales of *Lilium*; Lane 11: Loading buffer solution without RNA to serve as a control.


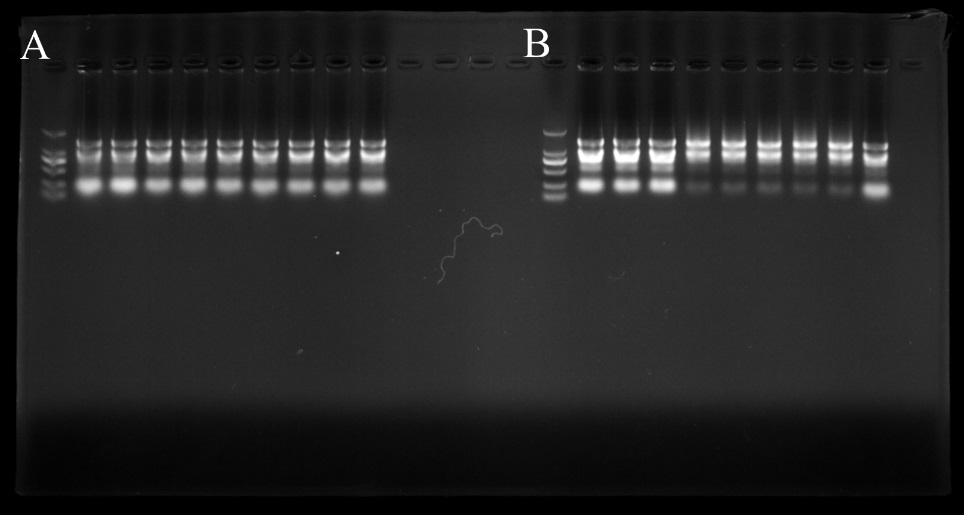


**Supplementary Figure 6. The original full-length gels used to display total RNAs in inner scales of *Lilium lancifolium* Thunb. and *Lilium brownii* var. *viridulum* Baker isolated using modified TRIzol method**. The order of samples of each gel lane is as follows: (A) Lane 1: DL2000 DNA marker; Lanes 2-4 are RNA isolated from top scales of *Lilium lancifolium* Thunb.; Lanes 5-7 are RNA isolated from middle scales of *Lilium lancifolium* Thunb.; Lanes 8-10 are RNA isolated from basal scales of *Lilium lancifolium* Thunb.; Lane 11: Loading buffer solution without RNA to serve as a control; (B) Lane 1: DL2000 DNA marker; Lanes 2-4 are RNA isolated from top scales of *Lilium brownii* var. *viridulum* Baker; Lanes 5-7 are RNA isolated from middle scales of *Lilium brownii* var. *viridulum* Baker; Lanes 8-10 are RNA isolated from basal scales of *Lilium brownii* var. *viridulum* Baker; Lane 11: Loading buffer solution without RNA to serve as a control*.*


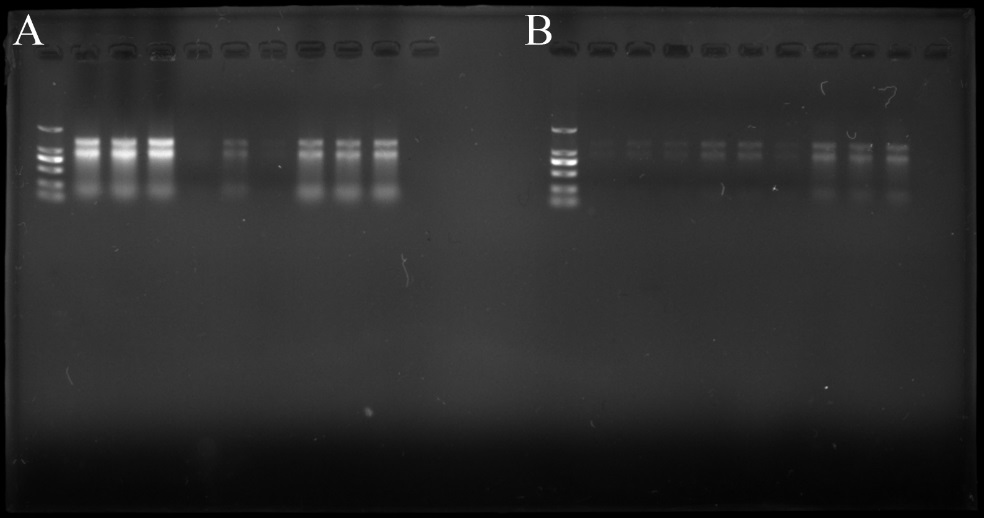


**Supplementary Figure 7. The original full-length gels used to display total RNAs in inner scales** **of *Lilium lancifolium* Thunb. and** ***Lilium brownii* var. *viridulum* Baker isolated using TRIzol method**. The order of samples of each gel lane is as follows: (A) Lane 1: DL2000 DNA marker; Lanes 2-4 are RNA isolated from top scales of *Lilium lancifolium* Thunb.; Lanes 5-7 are RNA isolated from middle scales of *Lilium lancifolium* Thunb.; Lanes 8-10 are RNA isolated from basal scales of *Lilium lancifolium* Thunb.; Lane 11: Loading buffer solution without RNA to serve as a control; (B) Lane 1: DL2000 DNA marker; Lanes 2-4 are RNA isolated from top scales of *Lilium brownii* var. *viridulum* Baker; Lanes 5-7 are RNA isolated from middle scales of *Lilium brownii* var. *viridulum* Baker; Lanes 8-10 are RNA isolated from basal scales of *Lilium brownii* var. *viridulum* Baker; Lane 11: Loading buffer solution without RNA to serve as a control*.*
